# Factors associated with the use of insecticide-treated nets: analysis of the 2018 Burkina Faso Malaria Indicator Survey

**DOI:** 10.1186/s12936-021-03756-5

**Published:** 2021-05-17

**Authors:** Mahamadi Tassembedo, Soumaila Coulibaly, Boukary Ouedraogo

**Affiliations:** 1 Institut de Formation et de Recherche Interdisciplinaires en Sciences de la Santé et de l’Education (IFRISSE), Ouagadougou, Burkina Faso; 2 Direction du Suivi, de l’évaluation et de la Capitalisation, Ministère de la santé, Ouagadougou, Burkina Faso; 3 Centre National de Recherche et de Formation sur le Paludisme (CNRFP)/Institut National de Santé Publique (INSP), Ministère de la santé, Ouagadougou, Burkina Faso; 4 Direction des systèmes d’information en santé, Ministère de la santé, Ouagadougou, Burkina Faso

**Keywords:** Burkina Faso, Malaria, Insecticide-treated nets, Malaria indicator survey

## Abstract

**Background:**

Sleeping under an ITN reduces contact with mosquitoes through the combination of a physical barrier and an insecticidal effect, which reduces the incidence of malaria. The 2016–2020 Burkina Faso National Malaria Strategic Plan aims to have at least 90% of the population, 100% of children under age 5, and 100% of pregnant women sleep under an ITN.

**Methods:**

The analysis examines individual, household, and community-level factors associated with ITN usage. According to the 2017–18 Burkina Faso MIS, 58% of individuals in households that own at least one ITN reported that they slept under an ITN on the night before the survey.

**Results:**

The use of ITNs was significantly associated with individual, household, and community-level variables that included age, gender, age of household head, number of sleeping rooms, wealth, malaria prevalence, residence, and region.

**Conclusions:**

The results highlight areas of intervention at the individual, household, and community levels that can increase ITN use.

## Background

Malaria is a disease caused by parasites that are transmitted to people though the bites of infected female *Anopheles* mosquitoes [1]. Malaria is a major cause of mortality and morbidity in developing countries. According to the World Health Organization (WHO), there were 229 million cases of malaria and 409,000 deaths from malaria worldwide in 2019, with 94% of cases and 94% of deaths occurring in the African Region [2].

One of the core malaria interventions recommended by the WHO to protect against mosquito bites is the use of insecticide-treated nets (ITNs). Sleeping under an ITN is effective in reducing the incidence of malaria by reducing contact with mosquitoes through the combination of a physical barrier and an insecticidal effect [3–5]. Across sub-Saharan Africa (SSA), there has been an increased focus on the scale-up and distribution of ITNs with the goal of having every household at risk of malaria transmission and every person within that household protected by an ITN [6, 7]. Countries have achieved high ITN coverage levels by using net distribution channels such as community delivery, routine health services, and outreach activities [8]. This investment in ITN distribution has increased the proportion of people in malaria-endemic areas sleeping under an ITN from 29% in 2010 to 50% in 2018 [2].

Studies have shown that the major driver of ITN use is access to an ITN [9–11]. However, individual, household, and community-level factors also influence ITN usage. At the individual level, factors that influence net use include age, gender, education, degree of control over household decision-making, ITN preferences, malaria knowledge and beliefs, and risk perception [12–17]. Household-level determinants of ITN include household size, household composition, number of sleeping rooms, and intra-household sleeping arrangements [11, 14, 18–21]. At the community level, factors such as residence, environmental conditions, and malaria seasonality have also been shown to influence ITN usage [9, 21, 22].

Burkina Faso implemented ITNs as the principal tool for malaria prevention. The 2016–2020 National Malaria Strategic Plan includes three approaches for ensuring that ITNs are available to the entire population: (1) free distribution of ITNs via nationwide campaigns, (2) free distribution of ITNs through routine antenatal care and expanded programmes on immunization at all public health facilities, and (3) the sale of ITNs by the private sector [23]. In 2011, the National Malaria Control Programme, in collaboration with its partners, implemented the first national campaign for the mass distribution of ITNs. This was followed by three other mass distribution campaigns in 2013, 2016, and 2019 [23]. To achieve universal ITN coverage, the national strategy aimed to provide enough ITNs to cover all residents of the household. The indicator that evaluates this strategy is the percentage of households with at least one ITN for every two people who stayed in the household the night before the interview. The percentage of households with at least one ITN for every two persons increased from 19% in the 2010 Burkina Faso Demographic and Health Survey (DHS) to 33% in the 2017–18 Burkina Faso Malaria Indicator Survey (MIS). Another focus of malaria prevention in Burkina Faso is the promotion of ITN use throughout the country. The 2016–2020 National Malaria Strategic Plan aims to have at least 90% of the population, 100% of children under age 5, and 100% of pregnant women in Burkina Faso sleep under an ITN [24].

**Fig. 1:**
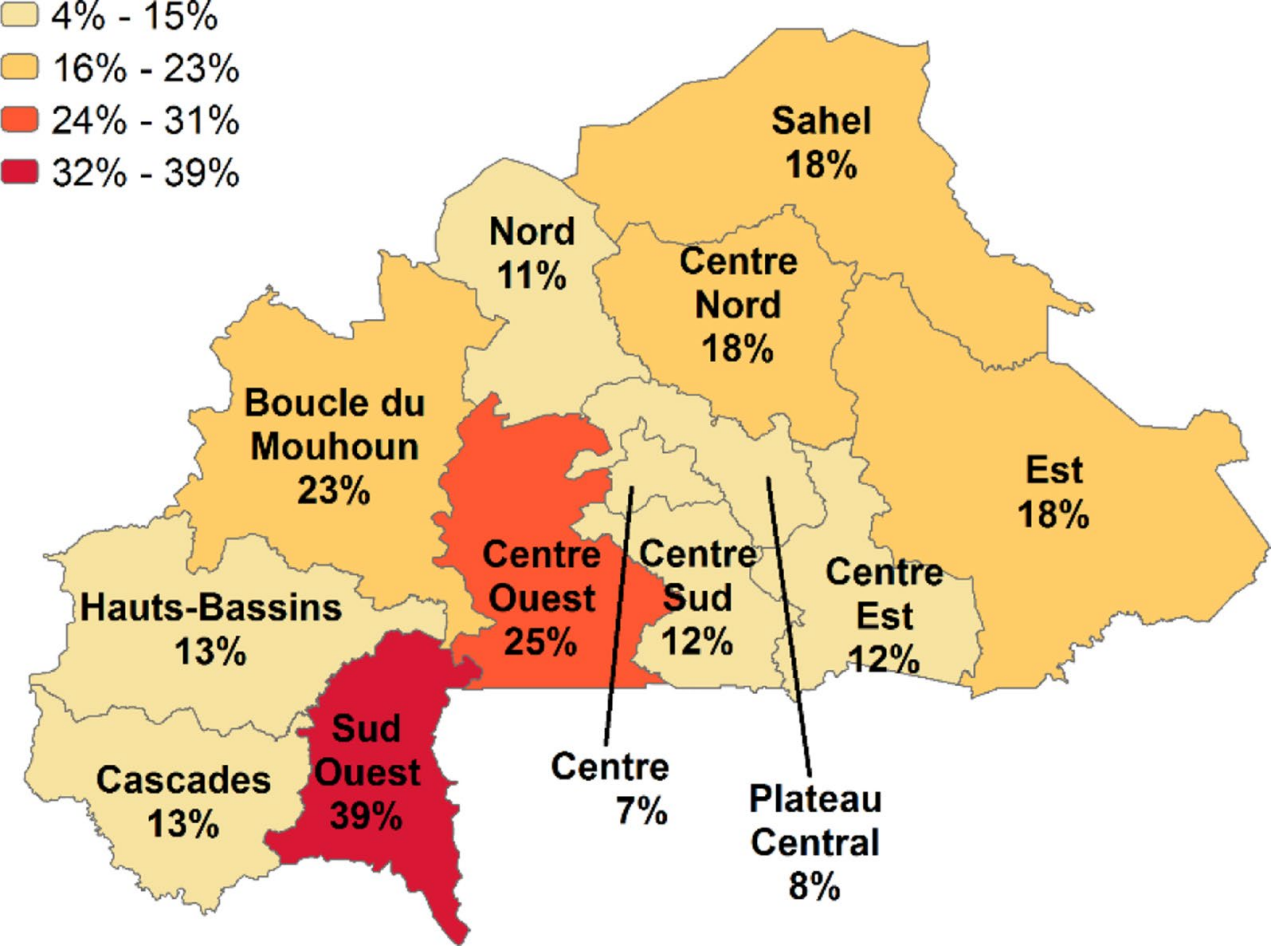
Percentage of children age 6–59 months who tested positive for malaria by microscopy

Burkina Faso is a landlocked Sahel country located in the centre of West Africa. Malaria remains a major public health issue and is endemic throughout the country [23]. The percentage of children age 6–59 months who tested positive for malaria by microscopy, according to the 2017–18 MIS, ranges from 7% in the Centre Region to 39% in the Sud Ouest Region (see Fig. 1). Malaria is seasonal across the country, with peak malaria season occurring from June through October. In Burkina Faso, the duration of the rainy season varies across the country with variances in seasonal malaria transmission based on geographic zones. In the north, the rainy season can last up to 3 months, while in the central zone it lasts up to 6 months, and in the south, 9 months [23].

The central research question is to determine the factors associated with the use of ITN nets in the population. This includes identifying high-risk areas in term of malaria prevalence, as well as factors at the individual, household, and community levels. Studying the factors that determine net use can lead to a better understanding of the actions that can increase net use in the general population and in specific areas of the country. These targeted actions will make it possible to improve the fight against malaria, which is the leading cause of mortality and morbidity in Burkina Faso. The results of this study will provide important answers on net use, and will inform decisions on distribution campaigns, the development of net replacement strategies, and the development and deployment of tools that include behaviour change communication activities.

## Methods

### Data

Data from the Burkina Faso 2017–18 MIS were used in the analysis. The MIS is a household survey of a representative sample of respondents. The MIS collected data on the availability and use of ITNs in households, and several other malaria indicators. External data were obtained from the Malaria Atlas Project (MAP) to determine the variable malaria prevalence in the analysis (described below). Since the Burkina Faso 2017–18 MIS collected GPS coordinates of the clusters, there was not possible to merge the external data with the DHS data at the cluster level.

### Variables

#### Outcomes variables

The dependent variable is a binary variable of whether an individual living in a household that owned at least one ITN had slept under an ITN the night before the survey. In addition, only de facto individuals that lived in households with at least one ITN were included in the analysis. This resulted in a sample size of 27,299 individuals or 27,333 after applying sample weights.

#### Independent variables

The choice of independent variables (or explanatory variables) was based on the data in the literature and was defined at the cluster, household, and individual level. One of the main variables of interest is malaria prevalence in 2015 estimated by the MAP. The variable is defined as the average parasite rate of *Plasmodium falciparum (PfPR)* in children between the ages of 2 and 10 within the 2 km (urban) or 10 km (rural) buffer that surrounds the DHS survey cluster location. This variable is used as a proxy to assess the malaria risk areas.

The remaining variables in the analysis include other cluster level variables of region and place of residence (urban/rural), that would also determine risk areas. The household level variables include wealth quintiles, number of household members (1–3, 4–6, 7–9, 10 +), number of sleeping rooms (0–1, 2, 3, 4, more than 4 rooms), and age of household head in years (less than 30, 30–39, 40–49, 50–59, and 60 +). The individual-level variables include age in years (0–10, 11–19, 20–29, 30–39, 40–49, 50 +), sex, and relationship to household head (wife/husband, son/daughter, and others).

The analysis took into account the availability of nets within households to determine the sample of net users. The population with “net access” was determined by estimating the proportion of the population that could be covered by existing ITN, assuming that each ITN in a household can be used by two people. This helps to assess the extent to which available ITN are used by individuals in households.

### Statistical analysis

This paper included descriptive analysis of the data, bivariate analysis to assess the association between the outcome and the independent variables, and multivariate logistic models to assess the magnitude of the associations after including controls. Three logistic models were fit to include each of the three cluster-level variables (malaria prevalence, region, and place of residence) separately. This is due to the high association of these three variables with one another. The multivariate models do not include the variables of number of household members and relationship to household head because these were highly correlated with the number of sleeping rooms and age of the individual, respectively. All analyses considered the survey sampling design and sampling weights, and used Stata version 16.

## Results

### Characteristics of the study population

Table 1 summarizes the characteristics of the study population. For the outcome variable, 58% of the sample reported that they slept under an ITN on the night before the survey. At the individual level, the sample includes a relatively young population with more than half (58%) of the sample under age 20. Most were either the son or daughter of the household head (53%). Most household heads were age 40–49 (27%) and age 30–39 (26%).

**Table 1:** Description of the de facto sample used in the analysis among households with at least one ITN

Variable	%	95% CI	N
Slept under an ITN last night			
Yes	58.4	[56.8, 60.0]	15,964
No	41.6	[40.0, 43.2]	11,369
Age in years			
0–10	34.6	[33.8, 35.5]	9464
11–19	23.6	[23.0, 24.3]	6456
20–29	13.0	[12.4, 13.6]	3539
30–39	11.7	[11.3, 12.2]	3208
40–49	7.1	[6.8, 7.4]	1927
50 +	10.0	[9.4, 10.6]	2731
Sex of household member			
Male	49.1	[48.5, 49.8]	13,430
Female	50.9	[50.2, 51.5]	13,903
Relationshop to household head			
Head	17.0	[16.6, 17.4]	4642
Wife/husband	18.5	[18.1, 18.8]	5045
Son/daughter	52.6	[51.7, 53.6]	14,384
Others	11.9	[11.0, 12.9]	3256
Age of household head			
Less than 30	8.9	[7.9, 10.1]	2444
30–39	25.6	[24.1, 27.2]	6991
40–49	27.3	[25.7, 28.9]	7460
50–59	18.5	[16.9, 20.2]	5054
60 +	19.7	[17.6, 21.9]	5371
Number of rooms for sleeping			
0–1 room	13.4	[12.2, 14.7]	3666
2 rooms	33.7	[31.8, 35.6]	9181
3 rooms	26.7	[24.8, 28.7]	7286
4 rooms	14.7	[13.2, 16.4]	4019
More than 4 rooms	11.5	[9.8, 13.4]	3128
Number of household members			
1–3	9.2	[8.4, 10.1]	2512
4–6	38.6	[36.7, 40.6]	10,556
7–9	29.0	[27.3, 30.7]	7923
10 or more	23.2	[21.0, 25.5]	6341
Wealth quintile			
Lowest	18.3	[16.0, 20.7]	4996
Second	20.1	[18.6, 21.6]	5493
Middle	20.6	[18.9, 22.4]	5623
Fourth	21.2	[19.2, 23.5]	5805
Highest	19.8	[17.6, 22.3]	5416
Malaria Prevalence^a^			
0–24%	9.1	[7.0, 11.8]	2270
25–34%	26.9	[21.9, 32.4]	6674
35–44%	38.7	[31.9, 45.9]	9617
45–60%	25.3	[20.0, 31.4]	6284
Place of residence			
Urban	18.5	[16.2, 21.1]	5053
Rural	81.5	[78.9, 83.8]	22,280
Region			
Boucle du mouhoun	12.1	[10.5, 13.9]	3310
Cascades	4.1	[3.3, 5.2]	1131
Centre	8.8	[7.6, 10.3]	2411
Centre est	6.9	[5.5, 8.5]	1877
Centre nord	8.8	[7.4, 10.3]	2398
Centre ouest	12.6	[8.8, 17.7]	3438
Centre sud	3.9	[3.3, 4.5]	1055
Est	8.3	[7.3, 9.4]	2266
Hauts-Bassins	11.2	[9.6, 13.1]	3068
Nord	9.2	[8.2, 10.3]	2513
Plateau Central	4.7	[4.1, 5.3]	1280
Sahel	6.2	[4.8, 8.1]	1702
Sud ouest	3.2	[2.7, 3.8]	885
Total			27,333

^a^Due to missing values, the total for this variable is 24,845

At the household level, most households had two or three rooms (34% and 27%, respectively) and between four to six household members (39%). The sample was predominantly rural (82%), from the regions of Centre Ouest (13%), Boucle du Mouhoun (12%), and Hauts-Bassins (11%). In terms of malaria prevalence, according to the 2015 MAP estimates, a quarter lived in areas with a prevalence of 45–60%, with the majority living in areas with a prevalence of 35–45% (39%). Only 9% of the sample lived in areas with a malaria prevalence of 0–24%.

According to the malaria prevalence variable, the total number of individuals is less than the total for the remaining variables. There were some clusters with missing information for this variable and this resulted in 2,478 unweighted individuals with missing values for this indicator.

### Bivariate analysis

The relationship between malaria prevalence, region, and place of residence was also examined. Table 2 shows that region and place of residence were both significantly associated with malaria prevalence. Areas of low malaria prevalence were more urban, with 37% of urban areas having a malaria prevalence of 0–24% compared to only 2% of rural areas with that prevalence. In addition, 84% of the Centre Region, which is highly urban, had a malaria prevalence of 0–24%. In contrast, 72% of the Hauts-Bassins Region was in the highest malaria prevalence category of 45–60%. This was followed by Boucle du Mouhoun (57%), Centre Nord (46%), and Nord (42%).

**Table 2:** Bivariate analysis of net use in households with at least 1 ITN for two people

Variable	%	95% CI	p-value
Age in years			< 0.001
0–10	61.2	[59.2, 63.2]	
11–19	44.9	[42.8, 47.0]	
20–29	64.2	[61.7, 66.6]	
30–39	66.8	[63.9, 69.5]	
40–49	63.6	[60.9, 66.2]	
50 +	59.6	[56.6, 62.5]	
Sex of household member			< 0.001
Male	53.6	[51.7, 55.4]	
Female	63.1	[61.3, 64.9]	
Relationship to household head			< 0.001
Head	62.3	[60.0, 64.6]	
Wife/husband	74.3	[72.3, 76.2]	
Son/daughter	53.8	[52.1, 55.6]	
Others	48.4	[45.1, 51.7]	
Age of household head			< 0.001
Younger than 30	72.3	[68.9, 75.4]	
30–39	63.8	[61.2, 66.3]	
40–49	57.1	[54.3, 59.8]	
50–59	55.2	[51.6, 58.7]	
60 +	50.0	[46.4, 53.5]	
Number of rooms for sleeping			< 0.001
0–1 room	71.3	[68.3, 74.1]	
2 rooms	63.0	[60.5, 65.4]	
3 rooms	55.2	[52.6, 57.7]	
4 rooms	51.6	[48.3, 54.9]	
More than 4 rooms	46.1	[42.0, 50.2]	
Number of household members			< 0.001
1–3	75.2	[72.0, 78.1]	
4–6	65.4	[63.2, 67.5]	
7–9	54.1	[51.2, 57.0]	
10 or more	45.6	[42.5, 48.7]	
Wealth quintile			0.001
Lowest	55.8	[52.7, 58.8]	
Second	57.2	[54.6, 59.6]	
Middle	57.4	[54.1, 60.6]	
Fourth	57.1	[54.1, 60.1]	
Highest	64.5	[60.9, 68.0]	
Malaria prevalence^a^			0.011
0–24%	67.3	[61.8, 72.4]	
25–34%	57.4	[54.2, 60.6]	
35–44%	55.8	[53.1, 58.5]	
45–60%	59.2	[54.8, 63.5]	
Place of residence			< 0.001
Urban	66.1	[62.8, 69.3]	
Rural	56.7	[54.8, 58.5]	
Region			< 0.001
Boucle du Mouhoun	58.9	[53.8, 63.8]	
Cascades	69.9	[65.8, 73.7]	
Centre	69.1	[63.9, 73.9]	
Centre Est	62.6	[57.2, 67.7]	
Centre Nord	63.4	[60.5, 66.1]	
Centre Ouest	50.7	[47.3, 54.0]	
Centre Sud	60.8	[53.3, 67.7]	
Est	61.0	[55.8, 66.0]	
Hauts-Bassins	58.3	[51.2, 65.0]	
Nord	61.6	[56.7, 66.3]	
Plateau Central	53.4	[46.6, 60.2]	
Sahel	39.8	[34.5, 45.3]	
Sud Ouest	45.4	[40.2, 50.7]	

^a^Due to missing values, the total for this variable is 24,845

Table 3 shows that all independent variables were significantly associated with the outcome. Individuals age 11–19 used ITNs the least (45%) compared to other age groups. More females (63%) slept under ITN nets compared to males (54%). At the household level, as the number of rooms or the number of household members increase, the use of ITN nets decreases. Households in the highest wealth quintile had the highest ITN use (65%), with the remaining wealth quintiles having similar rates of use between 56 and 57%.

**Table 3:** Crosstabulation of malaria prevalence with region and place of residence among households with at least one ITN

Variable	Malaria prevalence								p-value
	0–24%		25–34%		35–44%		45–60%		
	%	95% CI	%	95% CI	%	95% CI	%	95% CI	
Place of residence									< 0.001
Urban	37.4	[29.7, 45.8]	23.6	[16.9, 31.8]	31.8	[22.4, 43.0]	7.2	[1.9, 23.6]	
Rural	2.4	[1.1, 4.9]	27.6	[21.9, 34.3]	40.4	[32.4, 48.8]	29.6	[23.4, 36.8]	
Region									< 0.001
Boucle du Mouhoun	0.0		0.0		43.0	[21.3, 67.8]	57.0	[32.2, 78.7]	
Cascades	0.0		59.7	[38.0, 78.2]	37.0	[18.9, 59.7]	3.3	[0.4, 21.4]	
Centre	83.7	[59.3, 94.8]	16.3	[5.2, 40.7]	0.0		0.0		
Centre Est	0.0		100.0		0.0		0.0		
Centre Nord	0.0		20.2	[6.1, 49.6]	33.7	[14.4, 60.7]	46.1	[21.4, 72.8]	
Centre Ouest	0.0		15.1	[5.3, 36.4]	84.9	[63.6, 94.7]	0.0		
Centre Sud	33.9	[14.9, 60.0]	50.7	[27.8, 73.3]	15.4	[5.2, 37.6]	0.0		
Est	3.3	[0.4, 21.0]	34.8	[17.3, 57.8]	55.7	[32.6, 76.6]	6.1	[0.8, 34.4]	
Hauts-Bassins	0.0		0.0		28.4	[15.0, 47.1]	71.6	[52.9, 85.0]	
Nord	0.0		8.8	[1.2, 43.5]	49.0	[28.7, 69.6]	42.2	[22.9, 64.3]	
Plateau Central	6.2	[0.8, 35.1]	93.8	[64.9, 99.2]	0.0		0.0		
Sahel	0.0		52.6	[21.1, 82.2]	27.6	[8.1, 62.3]	19.7	[3.4, 63.5]	
Sud Ouest	0.0		0.0		70.5	[43.9, 88.0]	29.5	[12.0, 56.1]	
Total	9.1	[7.0, 11.8]	26.9	[21.9, 32.4]	38.7	[31.9, 45.9]	25.3	[20.0, 31.4]	

Use of ITN was higher in urban areas (66%) compared to rural areas (57%). In the MIS 2017–2018, the use of ITN was higher in the Cascade and Centre regions (around 60%) and lower in Sahel and Sud Ouest regions (respectively, 29 and 26%).. The prevalence of malaria in children 6–59 months is low in areas where ITN use is highest (Cascades and Centre).

### Multivariate analysis

Table 4 summarizes the adjusted odds ratios (AOR) estimates from the three models fitted to the outcome variable. Model 1 includes the malaria prevalence variable without region or place of residence. Model 2 includes the region variables without the malaria prevalence and place of residence variables, and Model 3 the place of residence includes variable without the malaria prevalence and region variables. All models had the same controls within each of the variables.

**Table 4:** Adjusted odds ratios for using ITNs for sleeping in households with at least one ITN for every two people

Variable	Model 1		Model 2		Model 3	
	AOR	95% CI	AOR	95% CI	AOR	95% CI
Age in years						
0–10 (Ref.)	1					
11–19	0.5***	0.5–0.6	0.5***	0.5–0.6	0.5***	0.5–0.6
20–29	0.9*	0.8–1.0	0.9*	0.8–1.0	0.9**	0.8–1.0
30–39	1.1*	1.0–1.3	1.1*	1.0–1.3	1.1*	1.0–1.3
40–49	1.2***	1.1–1.4	1.2***	1.1–1.4	1.2***	1.1–1.4
50 +	1.1	1.0–1.3	1.1	1.0–1.3	1.1	1.0–1.3
Sex of household member						
Male (Ref.)	1		1		1	
Female	1.5***	1.4–1.6	1.5***	1.4–1.6	1.5***	1.4–1.6
Age of household head						
Less than 30 (Ref.)	1		1		1	
30–39	0.7**	0.6–0.9	0.7**	0.6–0.9	0.7**	0.6–0.9
40–49	0.6***	0.5–0.8	0.7***	0.6–0.9	0.6***	0.5–0.8
50–59	0.7**	0.5–0.9	0.7*	0.6–0.9	0.7**	0.5–0.9
60 +	0.6***	0.4–0.7	0.6***	0.5–0.7	0.6***	0.4–0.7
Number of rooms for sleeping						
0–1 room (Ref.)	1		1		1	
2 rooms	0.7**	0.6–0.9	0.7***	0.6–0.8	0.8*	0.6–0.9
3 rooms	0.6***	0.5–0.7	0.5***	0.4–0.6	0.6***	0.5–0.7
4 rooms	0.5***	0.4–0.7	0.4***	0.3–0.6	0.5***	0.4–0.7
More than 4 rooms	0.4***	0.3–0.5	0.4***	0.3–0.5	0.4***	0.3–0.6
Wealth quintile						
Lowest (Ref.)	1		1		1	
Second	1.1	1.0–1.3	1.0	0.9–1.2	1.2	1.0–1.4
Middle	1.3*	1.0–1.5	1.2	1.0–1.4	1.3**	1.1–1.6
Fourth	1.3**	1.1–1.6	1.2*	1.0–1.4	1.3**	1.1–1.6
Highest	1.6***	1.2–2.0	1.4**	1.1–1.7	1.4**	1.1–1.8
Malaria prevalence						
0–24% (Ref.)	1		–	–	–	–
25–34%	0.7*	0.5–1.0	–	–	–	–
35–44%	0.7*	0.5–0.9	–	–	–	–
45–60%	0.8	0.6–1.2	–	–	–	–
Place of residence						
Urban (Ref.)	–	–	–	–	1	
Rural	–	–	–	–	0.8*	0.6–1.0
Region						
Boucle du mouhoun	–	–	0.7*	0.5–1.0	–	–
Cascades	–	–	0.9	0.7–1.3	–	–
Centre (Ref.)	–	–	1		–	–
Centre est	–	–	0.7	0.5–1.0	–	–
Centre nord	–	–	0.9	0.6–1.2	–	–
Centre ouest	–	–	0.5***	0.4–0.7	–	–
Centre sud	–	–	0.8	0.5–1.2	–	–
Est	–	–	0.8	0.6–1.2	–	–
Hauts-Bassins	–	–	0.6*	0.4–0.9	–	–
Nord	–	–	0.8	0.6–1.1	–	–
Plateau Central	–	–	0.5**	0.3–0.8	–	–
Sahel	–	–	0.3***	0.2–0.4	–	–
Sud ouest	–	–	0.4***	0.3–0.5	–	–

*Ref.* reference

*p < 0.05, **p < 0.01, ***p < 0.001

At the individual level, individuals age 11–19 had 50% lower odds of using an ITN compared to individuals age 0–10 in all three models. Those aged 40–49 had 20% greater odds of ITN use compared to individuals age 0–10. Females had 50% greater odds of using ITNs compared to males in all three models.

At the household level, heads of household age 30 and above had between 30 and 40% lower odds of using an ITN compared to heads of households younger than age 30. The odds of ITN use decrease with an increasing number of rooms. Households that have more than four rooms had 60% lower odds of ITN use compared to households with 0–1 rooms in all three models. In general, the odds of ITN use increases with increasing wealth quintile.

In Model 1, the odds of ITN use were significantly lower in areas with 25–34% and 35–44% malaria prevalence compared to areas with 0–24% prevalence (30% lower odds for both categories). In addition, there was no significant difference in ITN use between areas with the highest malaria prevalence of 45–60% and areas with the lowest prevalence of 0–24%.

In Model 2, individuals who live in the regions of Boucle du Mouhoun, Centre Ouest, Hauts-Bassins, Plateau Central, Sahel, and Sud Ouest had significantly lower odds of ITN use compared to the Centre Region. The highest disparities were found in Sahel and Sud Ouest with 70% and 60% lower odds, respectively, compared to the Centre Region.

In Model 3, individuals who live in rural areas had 20% lower odds of sleeping under an ITN net the night before the survey compared to individuals who live in urban areas.

## Discussion

Data from the Burkina Faso 2017–18 MIS were obtained from The DHS Program. This analysis evaluated factors that influence ITN use in Burkina Faso. Overall, 58% of individuals slept under an ITN in households that own at least one ITN. This proportion can be explained by the fact that the period of the survey (period of low malaria transmission) does not allow for a better appreciation of the actual use of ITNs by households. Other factors can also influence net use such as the absence of a net, use of a fan, and use of repellents or insecticide by households during the same night.

The ITN distribution campaign enabled the population to have at least one ITN for two people. Based on this availability, the main hypothesis of our study is that the use of these ITN by individuals is influenced by the context in which individuals operate, which can increase or decrease the behaviors of individuals when they are use ITNs to protect themselves from mosquito bites. These contextual factors are first and foremost individual; then individuals are influenced by households and finally by the community in which each individual lives.

The results showed differences in the use of nets at the individual, household, and community levels. At the individual level, older children had reduced odds of using an ITN as compared to the youngest children in the household. Females were also more likely to sleep under an ITN as compared to males. This corroborates previous research that found that most households in SSA prioritize ITN use in young children (children under age 5) and pregnant women of reproductive age. In these households, children share sleeping spaces with their mothers or with other female siblings [11, 20]. Older children are not priorities for nets, especially when a household does not have enough nets for all members in the household. One of the reasons for this policy choice is that older children who have been exposed to mosquito bites are likely to have contracted one or more malaria infections, so the perceived risk of infection for these children is lower than that of the youngest.

At the household level, the number of rooms for sleeping and wealth quintile were significantly associated with ITN use. The odds of ITN use decrease with an increasing number of rooms in households. This aligns with previous studies that found the main reasons for nonuse of ITNs to be lack of access to a net and not having enough nets for all household members within the household [10, 15, 25]. Typically, the bigger the household with more sleeping spaces, the more people live in the household. This analysis only examined ITN use in households with at least one ITN, and did not examine if the household had adequate nets for all the household members.

When examining ITN usage of the community level, ITN use was significantly associated with malaria prevalence, residence, and region. While these factors are interrelated, it does highlight that there are places within Burkina Faso that should be prioritized for future social behaviour messaging focused on increasing ITN usage. Previous studies have shown that over time, the Centre Region has consistently displayed high ITN usage, most likely reflective of the population in the Centre Region, which is concentrated in urban areas (Ouagadougou) [26]. This analysis aligns with prior research that found rural areas to have lower odds of ITN use as compared to urban areas, and all other regions in the country having lower odds of ITN usage as compared to the Centre Region. Finally, ITN use was significantly lower in areas with 25–34% and 35–44% malaria prevalence compared to areas with 0–24% prevalence. In addition, there was no significant difference in ITN use between areas with the highest malaria prevalence (45–60%) and areas with the lowest malaria prevalence (0–24%). This finding suggests the need to also emphasize other methods of malaria prevention (indoor spraying, larval nest control, use of mosquito netting in concessions) in medium and high prevalence areas in addition to promoting ITN use. Furthermore, a behavioral study on the under-use of ITN in these areas could shed more light on this finding.

There are several limitations to this study. First, the study period coincides with the low malaria transmission period in Burkina Faso. Past studies have shown that ITN use is highly influenced by variations in rainfall and malaria seasonality [22, 27]. This analysis controlled for malaria endemicity, but was unable to account for variations in rainfall and malaria seasonality across the country. Accurately controlling for patterns in seasonal malaria transmission requires access to more microlevel data, which were unavailable at the time of analysis. Future analysis should examine this in more detail. Secondly, this analysis did not control for intra-household characteristics that dictate ITN use. The intra-household characteristics of household members influences net allocation in households with too few nets to cover all household members [28]. Finally, the data analysed are cross-sectional in nature and thus do not permit causal inferences.

## Conclusion

Use of ITNs represents one of the most viable options for reducing malaria-related morbidity and mortality. The results of this study have highlighted areas of intervention at the individual, household, and community level that can increase ITN use. It is important to consider these factors in the national net distribution and awareness-raising strategies for malaria control in Burkina Faso. As the country works to eliminate malaria, the results of this study can help reduce the incidence of malaria and prevent its resurgence. In addition, periodic evaluation of malaria reduction strategies will provide a framework for reliably assessing the effectiveness of these interventions and informing future strategies that can eliminate malaria.

## Data Availability

Data and materials are available on DHS site: https://dhsprogram.com/data/available-datasets.cfm for MIS Burkina Faso 2017–18.
